# Machine learning analysis with population data for prepregnancy and perinatal risk factors for the neurodevelopmental delay of offspring

**DOI:** 10.1038/s41598-024-64590-8

**Published:** 2024-06-18

**Authors:** Seung-Woo Yang, Kwang-Sig Lee, Ju Sun Heo, Eun-Saem Choi, Kyumin Kim, Sohee Lee, Ki Hoon Ahn

**Affiliations:** 1 Research Institute of Medical Science, Konkuk University School of Medicine, Seoul, Republic of Korea; 2 School of Medicine, University of California, San Diego, USA; 3 AI Center, Korea University College of Medicine, Anam Hospital, Seoul, Korea; 4 Department of Pediatrics, Seoul National University Children’s Hospital, Seoul, Republic of Korea; 5 Department of Pediatrics, Seoul National University College of Medicine, Seoul, Republic of Korea; 6 Department of Statistics, Korea University College of Political Science and Economics, Seoul, Korea; 7 Graduate School of Artificial Intelligence, Pohang University of Science and Technology, Pohang, Korea; 8 Department of Obstetrics and Gynecology, Korea University College of Medicine, Anam Hospital, Seoul, Korea

**Keywords:** Risk factors, Paediatric research, Scientific data, Disability, Reproductive signs and symptoms

## Abstract

Neurodevelopmental disorders (NDD) in offspring are associated with a complex combination of pre-and postnatal factors. This study uses machine learning and population data to evaluate the association between prepregnancy or perinatal risk factors and the NDD of offspring. Population-based retrospective cohort data were obtained from Korea National Health Insurance Service claims data for 209,424 singleton offspring and their mothers who gave birth for the first time in 2007. The dependent variables were motor development disorder (MDD), cognitive development disorder (CDD) and combined overall neurodevelopmental disorder (NDD) from offspring. Seventeen independent variables from 2002 to 2007 were included. Random forest variable importance and Shapley Additive Explanation (SHAP) values were calculated to analyze the directions of its associations with the predictors. The random forest with oversampling registered much higher areas under the receiver-operating-characteristic curves than the logistic regression of interaction and non-linearity terms, 79% versus 50% (MDD), 82% versus 52% (CDD) and 74% versus 50% (NDD). Based on random forest variable importance, low socioeconomic status and age at birth were highly ranked. In SHAP values, there was a positive association between NDD and pre- or perinatal outcomes, especially, fetal male sex with growth restriction associated the development of NDD in offspring.

## Introduction

Neurodevelopmental disorders (NDDs) in offspring are associated with a complex combination of prenatal genetic factors or postnatal environment^1^. Delay in development is generally determined when a child does not attain developmental milestones compared to peers from the same population^2,3^. The terminology of developmental delay itself is not a definite diagnosis but rather an illustrative term with various definitions in the clinic^4^. However, one possible way to define developmental disorders can be “sequences that occur with delays or deviations in a child’s physical, cognitive, language or behavioral development”^3^. NDD includes motor developmental disorders (MDD) and cognitive developmental disorders (CDD) including attention-deficit/hyperactivity disorder (ADHD), autism spectrum disorder (ASD), learning disability (LD) and intellectual disability (ID)^5,6^.

The developmental origins of health and disease (DOHaD) theory is that various in utero environments during pregnancy induce predictive adaptive responses of offspring that anticipate later environments and that the degree of their adaptation between these environments and later environments is related to future disease risk^7,8^. The etiologies of NDDs vary, with both genetic and environmental factors being involved. Among the environmental factors, prepregnancy and perinatal factors are the most important^9,10^. Among the NDDs, ADHD and ASD are highly associated anatomical and genetical heritable factors. As is well known, levels of dopamine are different in people with ADHD than in those without ADHD^11^. Therefore, striatal dopamine transporter abnormalities are thought to underlie the pathophysiology and psychostimulant treatment^12^. ASD is highly associated with heritable factors such as epigenetic factors or genetic factors^13^. Cognitive disorder is associated with perinatal risk factors, including low birth weight, maternal body mass index (BMI) or maternal anemia^14–16^.

Despite the field of interest, there are few large cohort references that suggest perinatal risk factors for motor or cognitive and language developmental disorder. This study uses machine learning and population data to test the association between prepregnancy or perinatal risk factors and the neurodevelopmental disorders of offspring for as more reflective in the real world.

## Results

Descriptive statistics are shown in Table 1. NDD, including MDD and CDD, showed a higher tendency in the prepregnancy history of DM, HTN, and psychological problems. Other perinatal complications, such as PROM, placenta abruptio, GDM, PIH, PTB and antidepressant use history, are also increased in both MDD and CDD than normal. Model performance is presented in Table 2. The random forest with oversampling registered much higher AUCs than the logistic regression of interaction and non-linearity terms with oversampling, at 79% versus 50% (MDD), 82% versus 52% (CDD) and 74% versus 50% (NDD). Based on random forest variable importance, as shown in Table 3, low SES, age at birth, cesarean section, antidepressant use, prepregnancy depression, male fetus, prepregnancy anxiety, prepregnancy diabetes, prepregnancy hypertension, PIH and postpartum depression ranked within the top 10 for MDD, CDD and NDD.

**Table 1:** Baseline characteristics of the study population.

Variable	MDD (n = 6141)	CDD (n = 5434)	Typical development (n = 197,849)
Age mean	30.4 ± 3.5	30.1 ± 4.1	30.2 ± 3.8
SES mean	10.1 ± 5.1	9.7 ± 5.2	10.0 ± 5.0
Pregestational maternal disease			
HTN	144 (2.3)	134 (2.5)	3388 (1.7)
DM	237 (3.9)	228 (4.2)	5855 (3.0)
Depression	291(4.7)	317 (5.8)	7454 (3.8)
Anxiety	1147 (18.7)	915 (16.8)	28,864 (14.6)
Thyroid disease	37 (0.6)	22 (0.4)	989 (0.5)
Male neonate	3138 (51.1)	3677 (57)	99,543 (50.3)
PTB	1145 (18.6)	988 (18.2)	30,262 (15.2)
LGA	16 (0.3)	9 (0.2)	335 (0.2)
SGA	21 (0.4)	39 (0.7)	426 (0.2)
FGR	1254 (20.4))	1121 (20.6)	32,537 (16.4)
PROM	1158 (18.9)	999 (18.4)	30,660 (15.5)
Cesarean delivery	2438 (39.7)	2396 (44.1)	73,797 (37.3)
Placenta abruption	1191 (19.4)	1027 (18.9)	31,370 (15.9)
Postpartum depression	108 (1.8)	117 (2.2)	2410 (1.2)
GDM	496 (8.1)	424 (7.8)	12,555 (6.3)
PIH	75 (1.2)	80 (1.5)	1784 (0.9)
Antidepressant use	647 (10.5)	577 (10.6)	17,099 (8.6)

SES, social economic status; HTN, hypertension; DM. diabetes; PTB, preterm birth; LGA, large for gestational age; SGA, small for gestational age; FGR, fetal growth restriction; PROM, premature rupture of membrane; GDM, gestational diabetes; PIH, pregnancy induced hypertension.

**Table 2:** The areas under the receiver operating characteristic curve (AUC) for the random forest.

	MDD		CDD		NDD	
Logistic regression of interaction and non-linearity terms						
Accuracy	0.67		0.67		0.67	
AUC	0.50		0.52		0.50	
Sensitivity	0.67		0.67		0.67	
Specificity	0.46		0.56		0.50	
Confusion matrix	40,586	159	39,839	926	39,358	304
	20,103	137	19,271	1160	19,395	298
Random forest						
Accuracy	0.78		0.80		0.75	
AUC	0.79		0.82		0.74	
Sensitivity	0.80		0.82		0.77	
Specificity	0.73		0.73		0.70	
Confusion matrix	36,763	3993	36,455	4497	35,685	3951
	9471	10,758	7782	12,463	10,628	9091

**Table 3:** Random forest variable importance of prediction model.

Rank	MDD	Importance	CDD	Importance	NDD	Importance
1	SES	0.4115	SES	0.3771	Age	0.3999
2	Age	0.3859	Age	0.3219	SES	0.3902
3	Cesarean delivery	0.0409	Sex	0.1044	Sex	0.0441
4	Antidepressant	0.022	Cesarean delivery	0.0351	Cesarean delivery	0.0239
5	Pregestational depression	0.018	Pregestational anxiety	0.029	Antidepressant	0.0181
6	Pregestational anxiety	0.0157	Antidepressant	0.023	Pregestational anxiety	0.0172
7	Sex	0.0154	Pregestational DM	0.0139	Pregestational DM	0.0147
8	Pregestational DM	0.0149	Pregestational depression	0.0113	Pregestational depression	0.0117
9	Pregestational HTN	0.0123	Pregestational HTN	0.0095	Pregestational HTN	0.009
10	Postpartum Depression	0.0101	PIH	0.0082	PIH	0.0088
11	PIH	0.0098	Postpartum Depression	0.0081	Postpartum Depression	0.0086
12	GDM	0.0056	GDM	0.0072	GDM	0.0054
13	Thyroid disease	0.0055	FGR	0.0062	FGR	0.0047
14	FGR	0.0045	PTB	0.0044	PTB	0.0038
15	PTB	0.0039	Placenta abruptio	0.0037	Thyroid disease	0.0031
16	SGA	0.003	PPROM	0.0035	Placenta abruptio	0.003
17	Placenta abruptio	0.0028	SGA	0.0033	PROM	0.003
18	PPROM	0.0027	Thyroid disease	0.0027	SGA	0.0028
19	LGA	0.0026	LGA	0.0019	LGA	0.0022

SES, social economic status; HTN, hypertension; DM. diabetes; PTB, preterm birth; LGA, large for gestational age; SGA, small for gestational age; FGR, fetal growth restriction; PROM, premature rupture of membrane; GDM, gestational diabetes; PIH, pregnancy induced hypertension.

**Figure 1:**
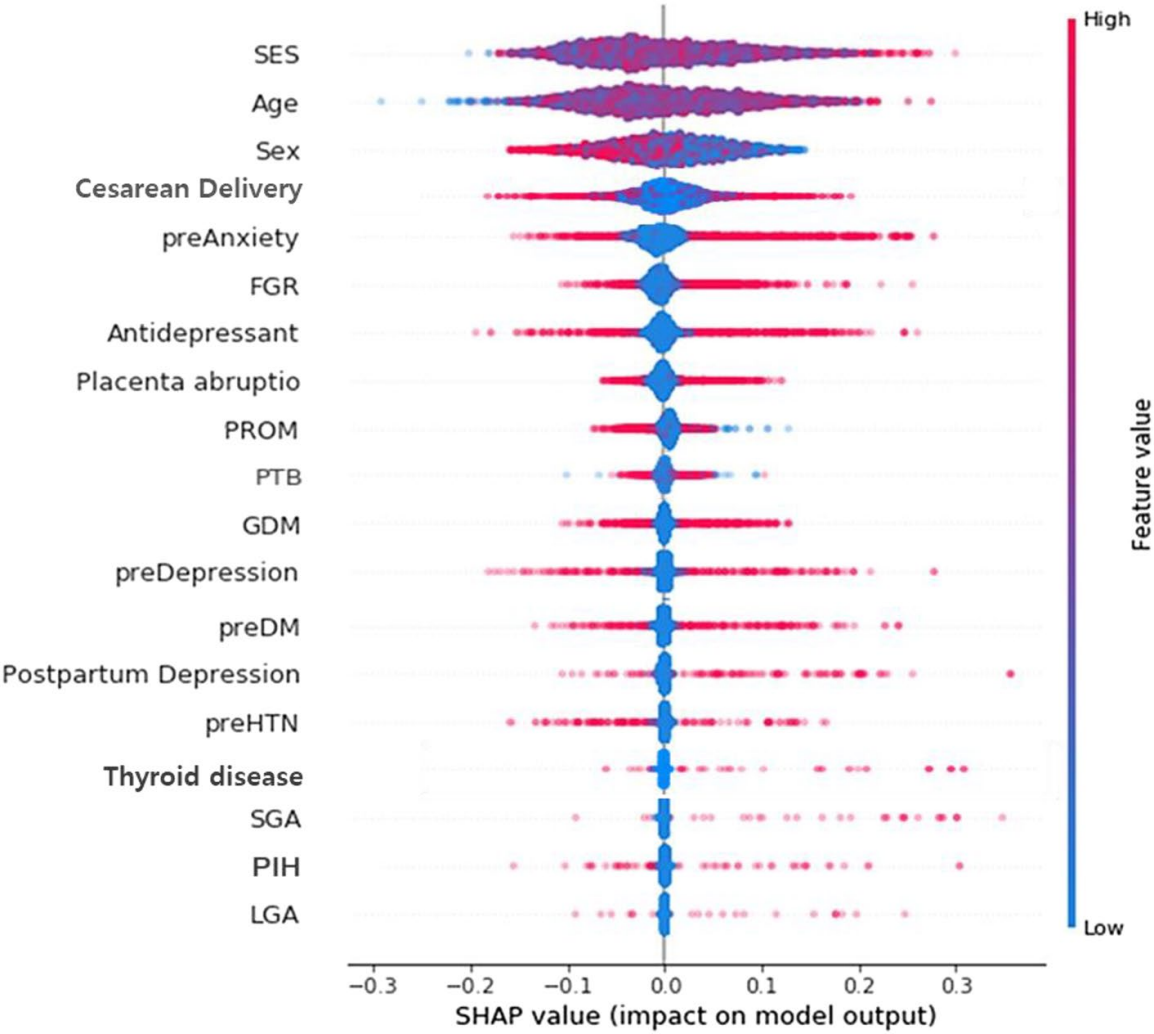
SHAP summary plot for MDD.

**Figure 2:**
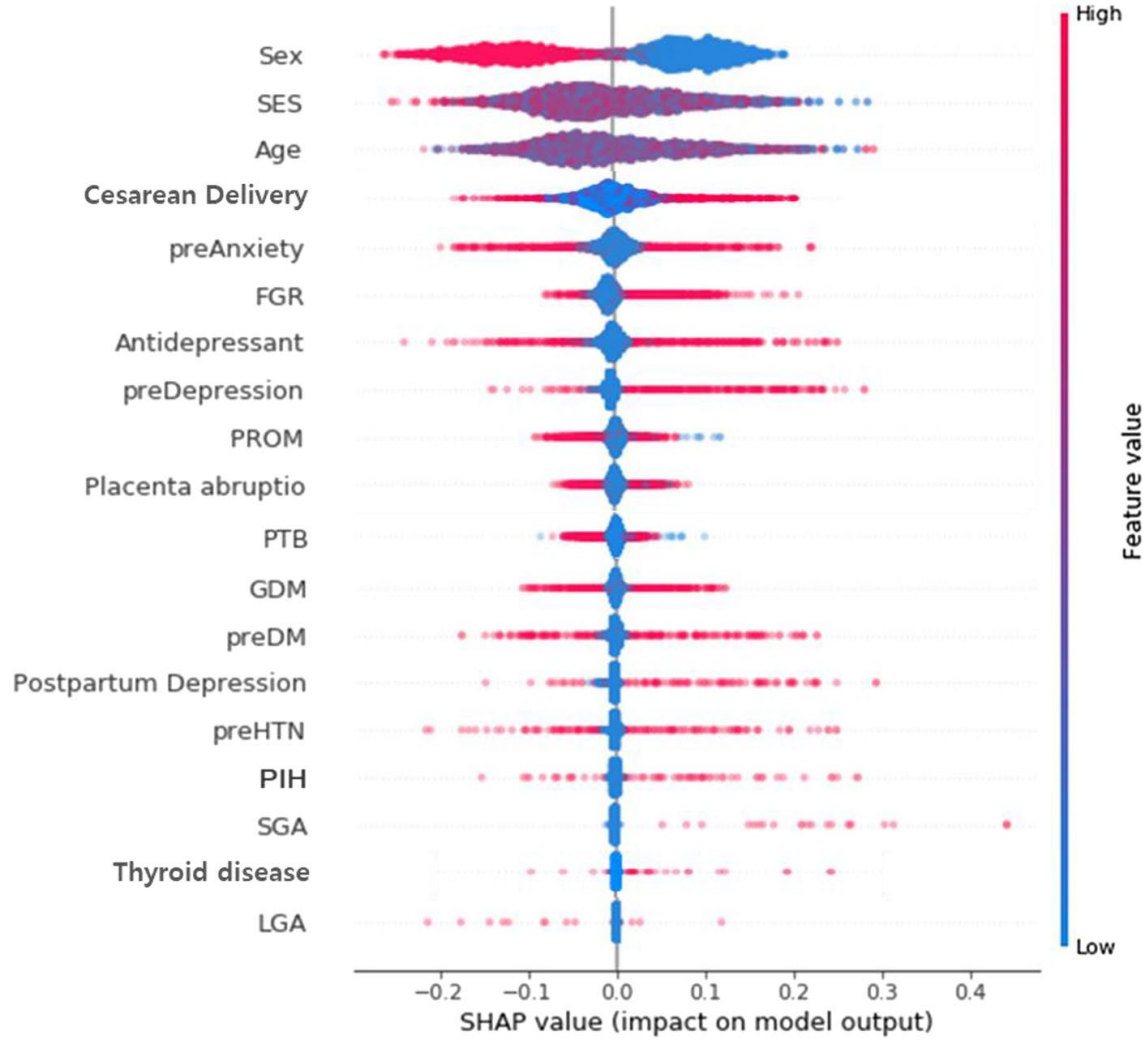
SHAP summary plot for CDD.

**Figure 3:**
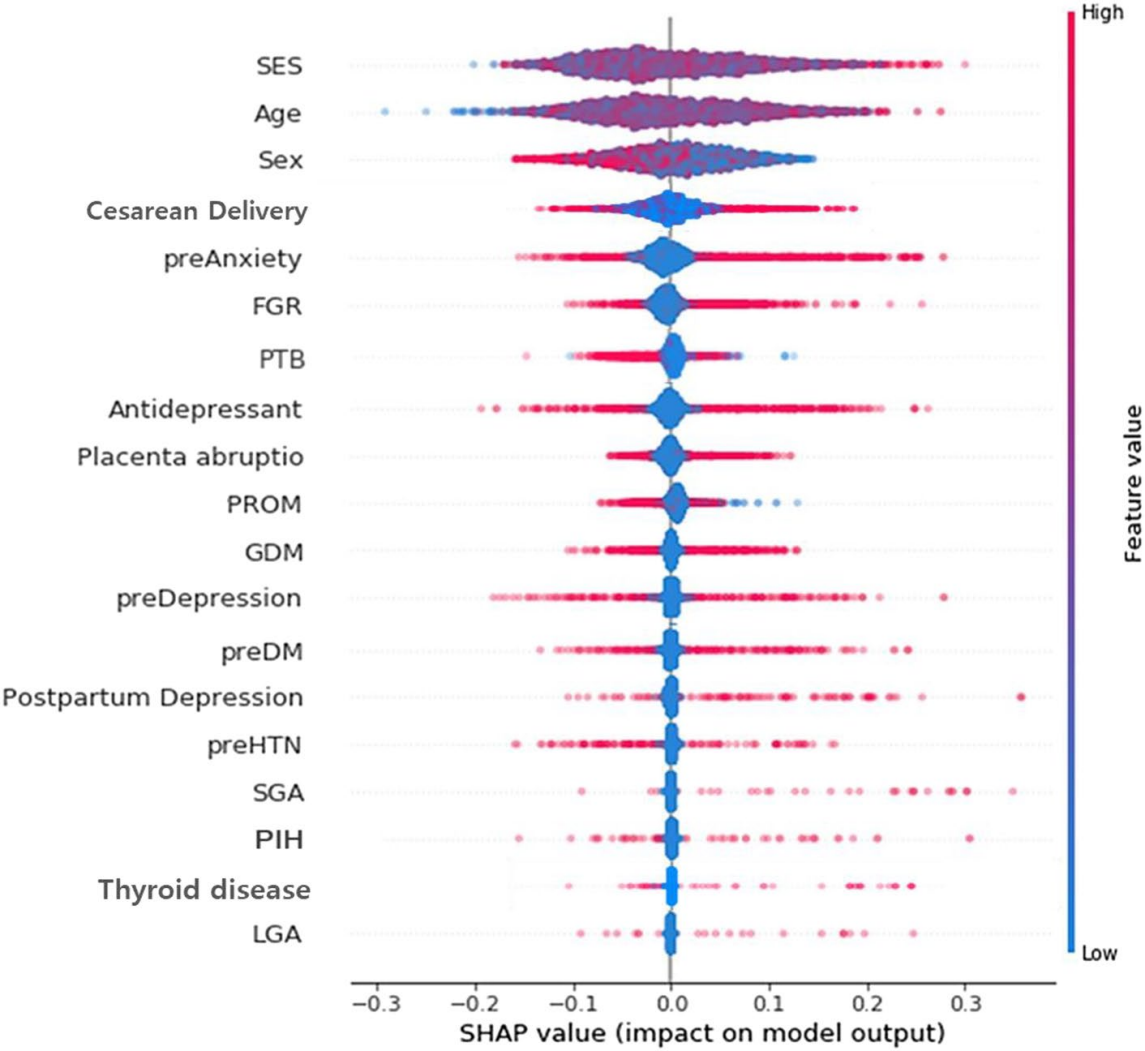
SHAP summary plot for NDD.

The positive association between NDD and its major predictor is more apparent from Shapley Additive Explanation (SHAP) value in Table S2. One way to evaluate the direction of association between neurodevelopment and its major predictor is to compare absolute values of max SHAP and min SHAP: (the former > the latter) denotes positive association and (the former < the latter) indicates negative association. For example, SHAP values of fetal growth restriction (FGR) for NDD have the range of (− 0.10, 0.27), some participants have SHAP values as low as − 0.10, and other participants have SHAP values as high as 0.27. This indicate that FGR into machine learning will decrease or increase the probability of the NDD by the range of − 0.10 to 0.27. Here, the absolute value of max SHAP (0.27) was greater than that of min SHAP (0.10). In other words, there exists a positive association between FGR and NDD in general. Figures 1, 2 and 3 are the SHAP summary plots for MDD, CDD and NDD, which plots the SHAP value of a major predictor for every participant. The blue (or red) color denotes the low (or high) value of a major predictor for a participant. For instance, in Fig. 3, blue points with the absence of FGR were located on the left side with low SHAP values, whereas red points with the presence of FGR were located on the right side with high SHAP values which are represented as − 0.10 to 0.27 (Table S2). The SHAP dependence plots, for every participant, the value of a predictor in the horizontal axis versus its SHAP value for in the vertical axis. In Figure S3, for instance, points with the absence of FGR (with a value of 0) were positioned in the left bottom with low SHAP values, while points with the presence of FGR (with a value of 1) were positioned in the right top with high SHAP values. Also, fetal male sex (the blue color) was positioned in the right top, therefore male sex is highest association with FGR for the prediction of NDD. However, the relationship between continuous variables and NDD can take a U-shaped form, as shown in Figure S3, such as social economic status (SES) and age.

## Discussion

In this study, we evaluated perinatal risk factors for offspring’s NDD with a higher accuracy model through random forest machine learning and SHAP variable importance analysis. As a result, maternal age and low social economic status most affected the development of NDD. Also, maternal risk factors, including psychological problems, pregnancy complications such as PIH and GDM, maternal prepregnancy DM, and fetal risk factors for FGR, small for gestational ages (SGA), and male sex, were associated with NDD. Additionally, higher-ranked important variables such as prepregnancy diabetes mellitus (DM)/hypertension (HTN), gestational diabetes mellitus (GDM), and pregnancy in hypertension (PIH) are very similar to previous literature that evaluated risk factors for NDD^17–20^.

The DOHaD theory suggests that the uncertain in-utero environment in early fetal developmental periods affected health risk factors in adulthood of offspring^7,8^. Based on this hypothesis, prediction, and identification of high-risk pregnancy groups for their offspring’s health, therefore, evaluate the preventive diagnosis, early intervention, and treatment for mothers^5^.

Age is a well-known risk factor for pregnancy complications. Both very young and advanced maternal age at childbirth affect the adverse outcomes of their offspring such as low birth weight and neonatal mortality^21,22^. Gao et al. reported that in terms of NDD, young and advanced maternal age at childbirth are associated with ADHD and LD risk^6^. In our results, age was one of the most important variables (Table 3) for the model and showed U-shape patterns in Figures S1, S2, and S3, which means that young and advanced ages were associated with the risk of MDD, CDD and NDD. Also, SES and age is the most associated factors.

Maternal psychological status and drug use also affected offspring NDD. Stress during pregnancy is also known to induce brain inflammation and influence fetal brain development^23^. It is well known that increased stress-related corticosteroid hormones such as cortisol and corticosterone are a consequence of stress. Fetal exposure to high concentrations of cortisol results in developmental delays and NDD^5^. Additionally, several researchers have reported that antidepressant drugs such as selective serotonin reuptake inhibitors (SSRIs) affect the development of ASD depending on whether disturbance of the serotonin system is involved in the pathophysiology of ASD^24,25^. In our results, maternal prepregnancy depression and anxiety history and antidepressant drugs are important risk factors for NDD development. In particular, anxiety and antidepressant drug use were highly positively correlated with NDD in the SHAP value analysis. Additionally, these factors are the most affected covariates to other variables in the SHAP independence plot. Maternal genetic liability also affects the offspring’s neurodevelopment and is related to pregnancy-related factors^26^. However, in this study, maternal genetic psychopathology was not defined, therefore, further study will be needed for this limitation.

In this study, fetal risk factors such as SGA, FGR, and male sex were associated with the development of NDD. Generally, FGR results in SGA and brain remodeling, in which the volume in the gray matter of the limbic region is reduced. In addition, the regional expanded volumes of the frontoinsular, frontal, and temporoparietal areas affect the disturbance of balanced neurodevelopment^27^. Additionally, male predominance in the incidence of NDD is often highlighted^28^. Females of many species including humans generally showed enhanced immune responses and increased resistance to disease and infection than males^29^. Because of several neurological disorders caused by pathological reactive microglia in central nervous system, sex difference in neurodevelopment is occurred^30^. Quinn et al. reported from a large-scale study that sex differences in reading impairment exist and are attributable largely to male vulnerability as opposed to ascertainment bias^31^. In our study, FGR and male sex were highly associated with a risk of NDD. Furthermore, in the dependence plot, FGR and male sex are strongly associated each other and covariates as risk factors for NDD. In their large cohort study of the relationship between low birth weight and LD at 11 years old, Johnson et al. reported that low birth weight was associated with an increased risk for LD in male offspring but not in female offspring (OR = 4.32, 95% CI 1.55–12.04). Additionally, these results depend on the difference in SES^15^. Within our study, SES, FGR, and male sex were highly ranked variables in the importance analysis, and this result reinforces the results of previous studies.

The limitation of this study is that it was a retrospective analysis utilizing an administrative database, which relies on the accuracy and consistency of the individuals coding the data. Therefore, the severity or grade of NDD was not fully adjusted. Additionally, due to limitations regarding the extraction of data on body mass index, adjustments for some well-known risk factors such as prepregnancy obesity were not performed^17,32^. Major issues of NDD, such as ADHD and ASD, were excluded in this cohort; therefore, these models have limitations in applications. However, as described above, because these two issues affect more genetic and heritable factors than other NDDs, these issues cause confounding bias in evaluating prepregnancy or pregnancy risk factors. In this study, age and SES are the two most affect factors in MDD, CDD, and NDD model. Therefore, there may be underestimated the influence of other variables respectively. For this, further subgroup analysis will be needed which adjusts the age and SES. FGR and preterm birth (PTB) are also known as major risk factors of NDD^33,34^. Especially, FGR, the early-on-set rather than late-on-set type is critically affected more severe patterns of neurodevelopmental outcome in offspring because of placenta insufficiency sequence^33,35^. Also, PTB in early gestational weeks in Influencing each other with FGR, which can be an important risk factor for NDD^34^. However, because our original coding data set does not identify gestational weeks of diagnosis for FGR and PTB, the influence of these factors may be underestimated. Notwithstanding the above limitations, this study had the advantage of involving a large nationwide assessment of the association between NDDs and various pregnancy risk factors with an accurate and high-validity machine learning model compared to the logistic regression of interaction and non-linearity terms. This study is expected to secure great validity and reliability based on population-based data with an unprecedented scale and the random forest with unusual performance.

## Methods

### Participants and variables

Almost 97% of the Korean population is enrolled in the Korea National Health Insurance (KNHI) sharing service program. Therefore, the KNHI claims database contains information on all claims for approximately 50 million Koreans, and nearly all information about the extent of a disease can be obtained from this centralized database. Population-based retrospective cohort data came from KNHI claims data for 209,424 singleton offspring and their primiparity mothers in 2007. This retrospective cohort study was approved by the Institutional Review Board (IRB) of Korea University Anam Hospital on 2022AN0184 (2022.04.11) and informed consent was waived by the IRB. Also, all methods were performed in accordance with the relevant guidelines and regulations. The dependent variables were MDD, CDD and NDD (sum of MDD and CDD) from 2007 to 2021 (Table S1). Seventeen independent variables were (1) five predictors in 2007, namely, age at birth (years), sex (male vs. female), low socioeconomic status [SES, measured by an insurance fee with a range of 1 (the highest group) to 20 (the lowest group)], small for gestational age (SGA), and large for gestational age (LGA); (2) four predictors from 2002 to 2006 (no vs. yes), namely, pregestational hypertension, pregestational diabetes, pregestational depression, and pregestational anxiety; (3) seven predictors within 10 months before childbirth (no vs. yes), namely, fetal growth restriction (FGR), premature rupture of membranes (PROM), placenta abruption, pregnancy induced hypertension (PIH), gestational diabetes (GDM), preterm birth (PTB) and antidepressant medication; and (4) one predictor within 12 months after childbirth, namely, postpartum depression. These predictors were screened from ICD-10 and anatomical therapeutic chemical (ATC) codes (N06A). (Table S1).

### Machine learning analysis

The logistic regression of interaction and non-linearity terms and the random forest were used for the prediction of NDD. Logistic regression employs a logistic function to estimate the probability of the dependent variable. Here, the outcome is 0 (or 1) for the probability below (or above) 0.5^36–38^. It needs to be noted that dependent variables of logistic regression in this study consist of four components with 57 terms: (1) 19 predictors listed in Table 1; (2) 18 interaction terms of Age multiplied by the other 18 variables (Age*SES + Age*Hypertension + Age*Diabetes + … + Age*Antidepressant); (3) 18 interaction terms of SES multiplied by the other 18 variables (SES*Age + SES*Hypertension + SES*Diabetes + … + SES*Antidepressant); two non-linearity terms (Age^2^, SES^2^). A random forest is a group of decision trees that make majority votes on the dependent variable (“bootstrap aggregation”). A random forest with 100 decision trees was performed. The training and testing of this random forest takes two steps. First, new data with participants are created based on random sampling with replacement, and a decision tree is created based on these new data. Here, some participants in the original data are excluded from the new data, and these leftovers are called out-of-bag data. This process is repeated 100 times; specifically, 100 new data are created, 100 decision trees are created, and 100 out-of-bag data are created. Second, the 100 decision trees make predictions on the dependent variable of every participant in the out-of-bag data, their majority vote is taken as their final prediction on this participant, and the out-of-bag error is calculated as the proportion of wrong votes on all participants in the out-of-bag data^36–38^. In this study total 209,424 cases with full information were split into training and validation sets at an 80:20 ratio (167,539 vs. 41,885 cases). A criterion for the validation of the trained models (i.e., logistic regression and random forest) was accuracy (a ratio of correct predictions among 41,885 cases) and the area under the receiver-operating-characteristic curve (AUC) (area under the plot of sensitivity vs. 1—specificity).

### Variable importance analysis

SHAP values were calculated to analyze the directions of association between NDD and predictors in the prediction model. R-Studio 1.3.959 (R-Studio Inc: Boston, United States) was employed for the analysis during January 1, 2023–February 28, 2023. The SHAP value of a predictor for a participant measures the difference between what machine learning predicts for the probability of MDD, CDD and NDD with and without the predictor. Random forest impurity importance and random forest permutation importance were the only explainable artificial intelligence methods before machine learning accuracy importance, and SHAP was introduced as an extension or alternative very recently. In other words, the SHAP value combines the results of all possible subgroup analyses, which are ignored in linear or logistic regression with an unrealistic assumption of *ceteris paribus*, namely, “all the other variables staying constant”^36–38^.

### Supplementary Information

Supplementary Figures.

Supplementary Tables.

## Data Availability

All data generated or analysed during this study are included in this published article and its supplementary information files. The datasets generated and/or analysed during the current study are not publicly available. This is because the dataset for this study is only available on the NHIS servers for 1 year after the dataset was generated.
