# Supplementary material for: Machine learning analysis with population data for prepregnancy and perinatal risk factors for the neurodevelopmental delay of offspring
Source: Sci Rep. 2024 Jun 18;14:13993. doi: 10.1038/s41598-024-64590-8 (PMC11183197; doi:10.1038/s41598-024-64590-8)

Figure S1. SHAP Dependence Plots: MDD

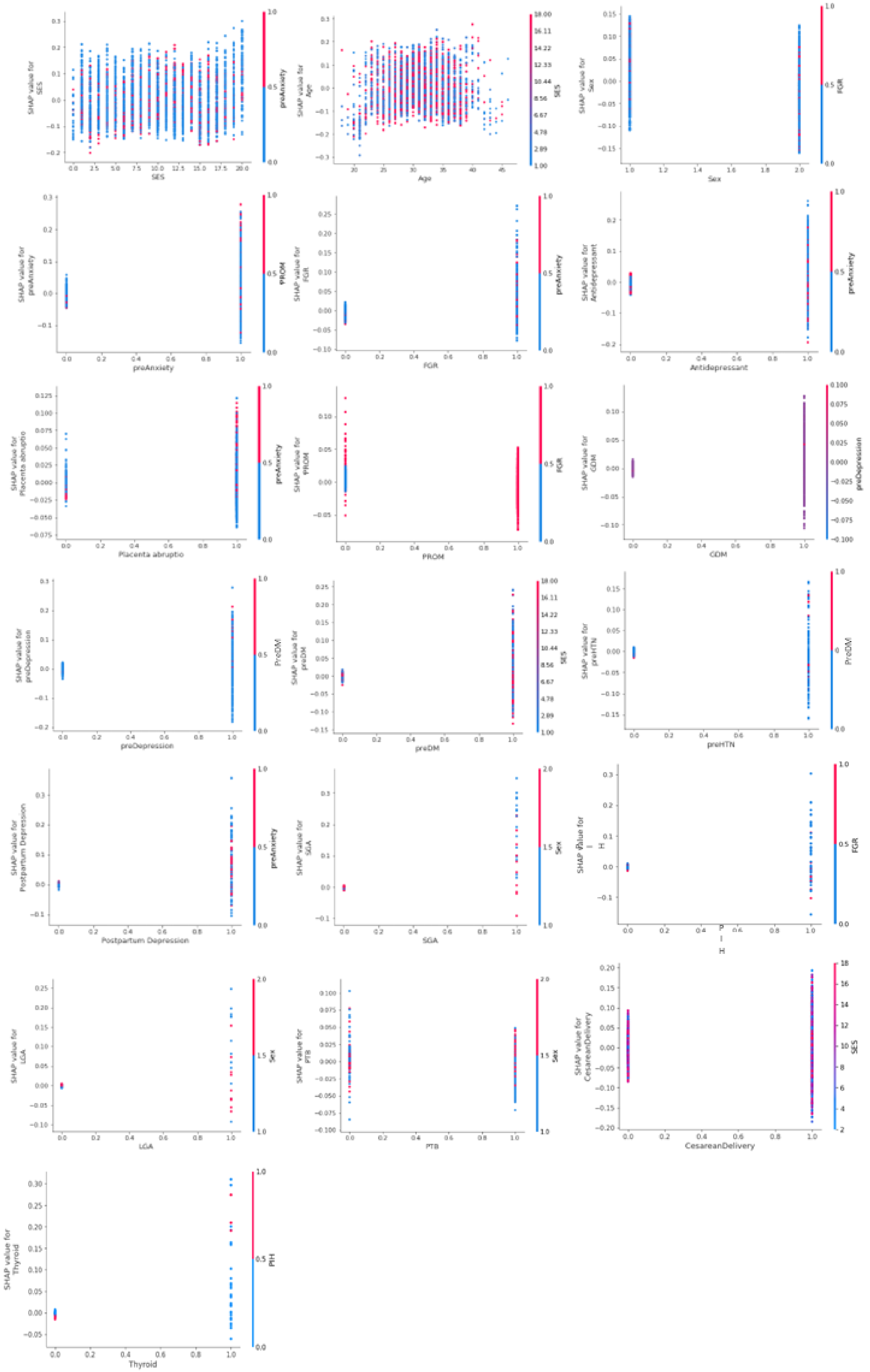

Figure S2. SHAP Dependence Plots: CDD

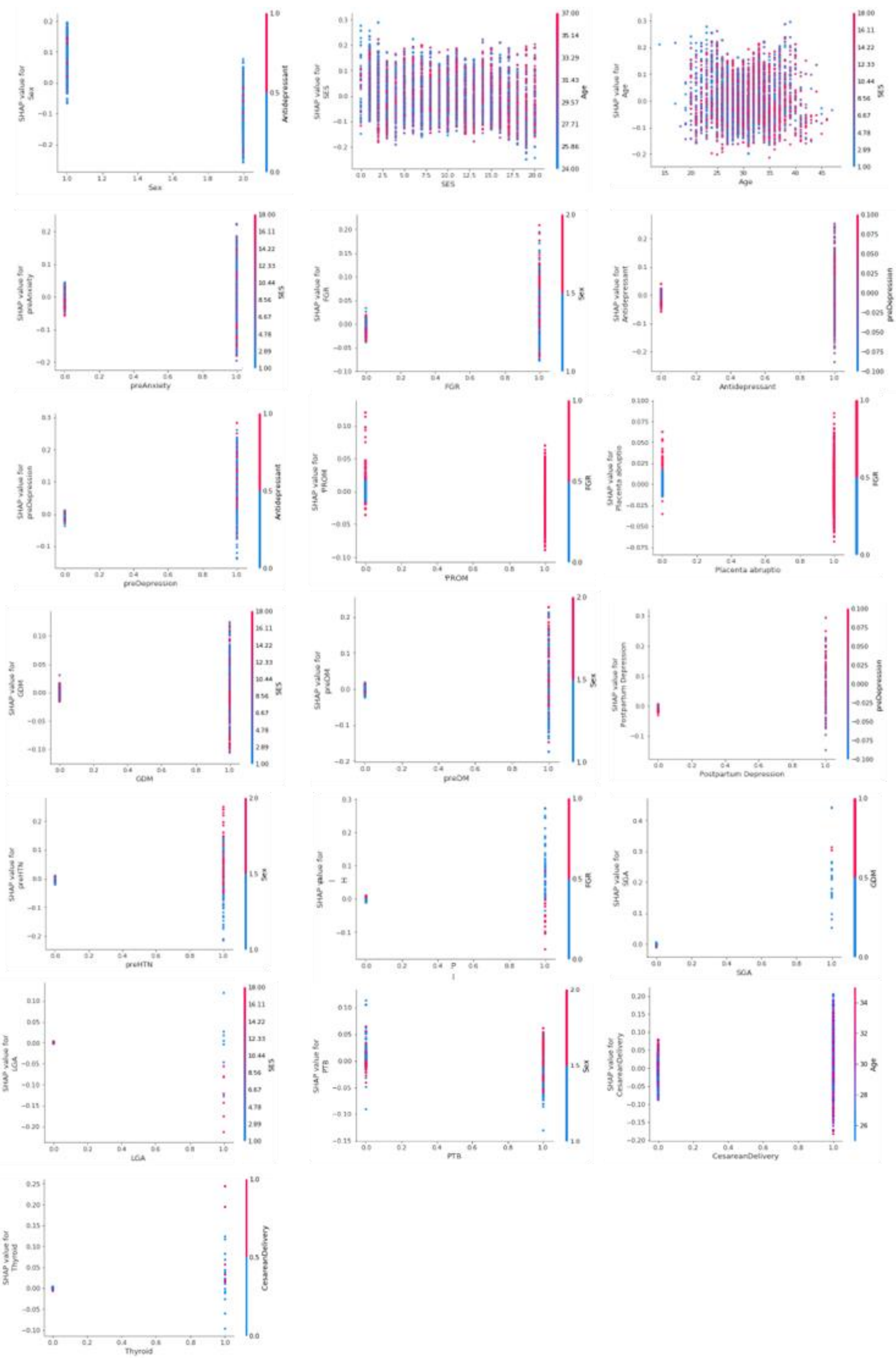

Figure S3. SHAP Dependence Plots: NDD

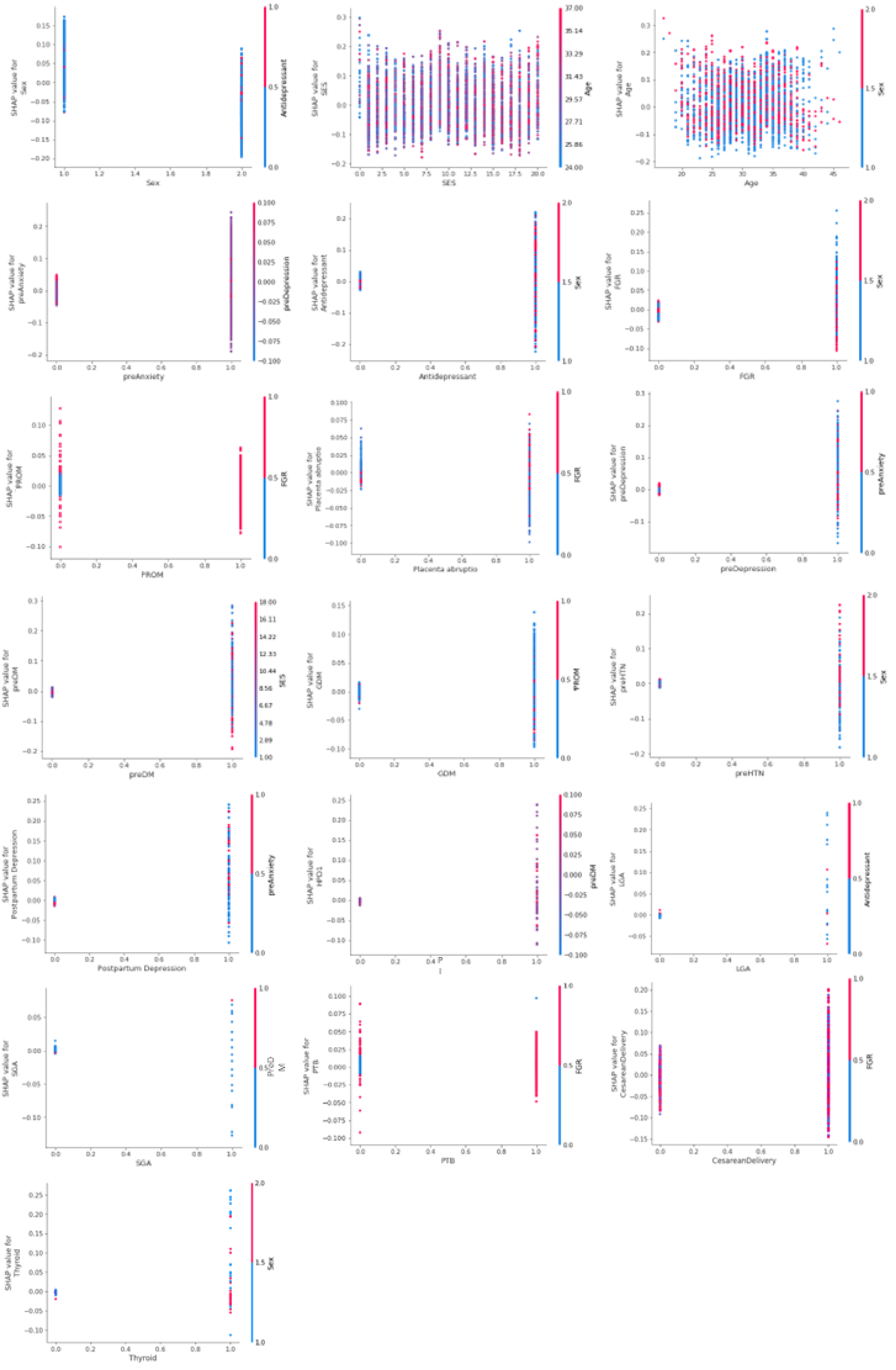

Supplement: Supplementary file 1 — Supplementary Figures. [file 41598_2024_64590_MOESM1_ESM.pdf]
