# Supplementary material for: Machine learning analysis with population data for prepregnancy and perinatal risk factors for the neurodevelopmental delay of offspring
Source: Sci Rep. 2024 Jun 18;14:13993. doi: 10.1038/s41598-024-64590-8 (PMC11183197; doi:10.1038/s41598-024-64590-8)
Supplement: Supplementary file 2 — Supplementary Tables. [file 41598_2024_64590_MOESM2_ESM.docx]

S1 Table. ICD-10 code using for variables

| Varable | CODE | Description |
| --- | --- | --- |
| Motor developmental disorder | F82 | Specific developmental disorder of motor function |
|  | R26 | Abnormalities of gait and mobility |
|  | R27 | Other lack of coordination |
| Cognitive developmental disorder | F70-F79 | Mental retardation |
|  | F80 | Specific developmental disorders of speech and language |
|  | F81 | Specific developmental disorders of scholastic skills |
|  | F83 | Mixed specific developmental disorders |
|  | R48.0 | Dyslexia and alexia |
|  | R48.8 | Other and unspecified symbolic dysfunctions |
| Fetal growth restriction | O36.5 | Maternal care for poor fetal growth |
| SGA | P05.1 | Small for gestational age |
|  | P05.2 | Fetal malnutrition without mention of light or small for gestational age |
| LGA | P08.1 | Other heavy for gestational age infants |
| PROM | O42.0 | Premature rupture of membranes, onset of labour within 24 hours |
|  | O42.1 | Premature rupture of membranes, onset of labour after 24 hours |
|  | O42.2 | Premature rupture of membranes, labour delayed by therapy |
|  | O42.9 | Premature rupture of membranes, unspecified |
| Placenta abruptio | O45 | Premature separation of placenta [abruptio placentae] |
| Pregestational HTN | I10-I15 | Hypertensive diseases |
| Pregestational DM | E10-E14 | Diabetes mellitus |
|  | O24.0 | Pre-existing type 1 diabetes mellitus |
|  | O24.1 | Pre-existing type 2 diabetes mellitus |
|  | O24.2 | Pre-existing malnutrition-related diabetes mellitus |
|  | O24.3 | Pre-existing diabetes mellitus, unspecified |
| Pregestational depression | F32 | Depressive episode |
|  | F33 | Recurrent depressive disorder |
|  | F34 | Persistent mood [affective] disorders |
|  | F38 | Other mood [affective] disorders |
|  | F39 | Unspecified mood[affective] disorder |
| Pregestational anxiety | F40 | Phobic anxiety disorders |
|  | F41 | Other anxiety disorders |
|  | F44 | Dissociative [conversion] disorders |
|  | F45 | Somatoform disorders |
|  | F48 | Other neurotic disorders |
| Postpartum Depression | F32 | Depressive episode |
|  | F33 | Recurrent depressive disorder |
|  | F34 | Persistent mood [affective] disorders |
|  | F38 | Other mood [affective] disorders |
|  | F39 | Unspecified mood[affective] disorder |
|  | F53 | Mental and behavioural disorders associated with the puerperium, NEC |
| PIH | O13 | Gestational [pregnancy-induced] hypertension |
|  | O14 | Pre-eclampsia |
|  | O15 | Eclampisa |
|  | O16 | Unspecified maternal hypertension |
| GDM | O24.4 | Diabetes mellitus arising in pregnancy |
|  | O24.9 | Diabetes mellitus in pregnancy, unspecified |
| PTB | O42.0 | Premature rupture of membranes, onset of labour within 24 hours |
|  | O42.1 | Premature rupture of membranes, onset of labour after 24 hours |
|  | O42.2 | Premature rupture of membranes, labour delayed by therapy |
|  | O42.9 | Premature rupture of membranes, unspecified |
|  | O60.1 | Preterm spontaneous labour with preterm delivery |
|  | O60.3 | Preterm delivery without spontaneous labour |
| Thyoid desease | E05 | Thyrotoxicosis (Hyperthyroidism) |
|  | E03 | Hyporthyroidism |

HTN, hypertension; DM. diabetes; PTB, preterm birth; LGA, large for gestational age; SGA, small for gestational age; FGR, fetal growth restriction; PROM, premature rupture of membrane; GDM, gestational diabetes; PIH, pregnancy induced hypertension

S2 Table SHAP value of the prediction model

| Variable | MDD | | CDD | | NDD | |
| --- | --- | --- | --- | --- | --- | --- |
|  | Min | Max | Min | Max | Min | Max |
| SES | -0.19256 | 0.29056 | -0.19256 | 0.29056 | -0.19256 | 0.29056 |
| Age | -0.26403 | 0.25738 | -0.26403 | 0.25738 | -0.26403 | 0.25738 |
| Sex | -0.16774 | 0.14717 | -0.16774 | 0.14717 | -0.16774 | 0.14717 |
| Pregestational HTN | -0.17321 | 0.16813 | -0.17321 | 0.16813 | -0.17321 | 0.16813 |
| Pregestational DM | -0.13992 | 0.24366 | -0.13992 | 0.24366 | -0.13992 | 0.24366 |
| Pregestational Depression | -0.16814 | 0.28047 | -0.16814 | 0.28047 | -0.16814 | 0.28047 |
| Pregestational Anxiety | -0.16468 | 0.28672 | -0.16468 | 0.28672 | -0.16468 | 0.28672 |
| LGA | -0.07380 | 0.26823 | -0.07380 | 0.26823 | -0.073801 | 0.26823 |
| SGA | -0.12090 | 0.35875 | -0.12090 | 0.35875 | -0.12090 | 0.35875 |
| FGR | -0.10489 | 0.27018 | -0.10489 | 0.27018 | -0.10489 | 0.27018 |
| PROM | -0.06737 | 0.08073 | -0.06737 | 0.08073 | -0.06737 | 0.08073 |
| Placenta abruptio | -0.06257 | 0.10826 | -0.06257 | 0.10826 | -0.06257 | 0.10826 |
| Postpartum Depression | -0.09663 | 0.35204 | -0.09663 | 0.35204 | -0.09663 | 0.35204 |
| GDM | -0.11983 | 0.12428 | -0.11983 | 0.12428 | -0.11983 | 0.12428 |
| PIH | -0.13213 | 0.29380 | -0.13213 | 0.29380 | -0.13213 | 0.29380 |
| Antidepressant | -0.17970 | 0.25439 | -0.17970 | 0.25439 | -0.17970 | 0.25439 |
| PTB | -0.09230 | 0.09726 | -0.09230 | 0.09726 | -0.09230 | 0.09726 |
| Cesarean delivery | -0.18514 | 0.19334 | -0.18238 | 0.204882 | -0.14571 | 0.200784 |
| Pregestational  Thyroid disease | -0.06113 | 0.31054 | -0.09667 | 0.244367 | -0.11271 | 0.262443 |

Abbreviation: SES, social economic status; HTN, hypertension; DM. diabetes; PTB, preterm birth; LGA, large for gestational age; SGA, small for gestational age; FGR, fetal growth restriction; PROM, premature rupture of membrane; GDM, gestational diabetes; PIH, pregnancy induced hypertension
